# Supplementary material for: Are carbon emissions trading and green financial instruments synergistic? -Comprehensive quantitative research based on content analysis
Source: PLoS One. 2024 Mar 7;19(3):e0298601. doi: 10.1371/journal.pone.0298601 (PMC10919601; doi:10.1371/journal.pone.0298601)
Supplement: S1 Data — (ZIP) [file pone.0298601.s001.zip › Data/地级市Z_F工作报告.zip!/╝¬┴╓/│ñ┤║╩╨/└┤╫╘╣1⁄2╓┌║┼í╛┬φ┐╦╩2╛▌═°í┐_2006.docx]

各位代表：

　　我代表市人民政府向大会作工作报告，请予审议。并请列席会议的同志提出意见。

　　一、“十五”和2005年工作简要回顾

　　“十五”时期，在省委、省政府和市委的领导下，在市人大、政协的监督支持下，我们紧紧依靠全市人民，成功战胜非典疫情和各种自然灾害的挑战，从容应对加入世贸组织后的新变化，全力振兴长春老工业基地，全面完成了“十五”计划，改革开放和现代化建设跃上一个新台阶。

　　——经济持续快速增长，综合实力明显增强。主要经济指标实现或接近翻番。预计2005年全市地区生产总值完成1675亿元，比2000年增长近一倍，年均增长12.5%。全口径财政收入实现184.8亿元，增长1.4倍，年均增长16.5%。规模以上工业总产值实现1712亿元，增长1.3倍，年均增长18%。外贸进出口总额完成44亿美元，增长1.6倍，年均增长20.8%。社会消费品零售总额实现600亿元，增长0.9倍，年均增长14%。“十五”期间，全社会固定资产投资累计完成2105亿元，比“九五”时期增长1.6倍。规模以上工业企业累计实现利润353.3亿元，增长2.3倍。累计实际利用外资39.6亿美元，增长2.2倍。累计引进内资750.3亿元，增长8.4倍。

　　——结构调整取得明显成效，经济整体素质进一步提升。产业结构不断优化，一、二、三产业比例由“九五”时期的14.3：43.2：42.5调整到10.7：46.8：42.5。粮食产量稳定在150亿斤的阶段性水平，畜牧业占农业总产值的比重达到54.2%。工业整体素质明显提高，汽车产能和农产品加工能力分别达到100万辆、720万吨，全市产值超亿元和超10亿元的企业分别发展到113户和13户。国家光电子产业基地、生物产业基地启动建设，高新技术产值占工业总产值的比重由19.3%提高到46%。物流业、房地产业、旅游会展业快速发展，现代服务业占服务业的比重由29%提高到43%。民营经济不断壮大，混合型经济快速成长，非国有经济占地区生产总值的比重由50%提高到60.2%。

　　——各项改革扎实推进，对外开放持续扩大。基本完成市属国有工商企业改革，558户国有企业实现产权多元化。实施了以“一免三补”为主要内容的农村税费改革,粮食流通体制改革稳步推进，投融资体制、住房制度等项改革逐步深入，市场在资源配置中的基础性作用不断增强。以养老、失业、基本医疗保险为重点的社会保障体系日益完善，涵盖生活费发放以及住房、医疗、教育、供热等城市低保救助体系初步形成。对外开放向广度和深度扩展，在长投资的世界500强企业由15户增至40户，引进投资超千万美元的项目295个，新批“三资企业”613家，外贸出口企业由200户增加到356户，出口市场由70个国家和地区拓展到140个。对外交流合作更加密切，与30多个国家和地区的50多个城市建立了友好关系。开发区经济占全市经济的比重达到51.3%，实现了2000年提出的“再造一个长春”的目标。

　　——城市建设步伐加快，环境质量明显改善。累计投入500多亿元，实施了引松二期、石头口门水库增容、龙嘉国际机场、长双烟铁路、轻铁、伊通河整治、道路建设、巷道改造、供热和供水管网建设等一大批城建重点工程，基础设施实现从严重制约到基本适应的根本性转变。通过实施新的城市管理体制、“畅通工程”和市容环境治理，城市管理水平不断提高。城区实现自来水普及率96%，综合气化率95.9%，集中供热热化率75%，生活垃圾无害化处理率83.5%，工业固体废旧物综合利用率97%，粪便无害化处理率75%，建成区绿化覆盖率41.5%，城市空气环境质量优良天数340天以上，城区道路完好率90%，88%的行政村实现通油（水泥）路。

　　——群众基本利益得到保障，人民生活质量不断提高。累计开发就业岗位40万个，新安置就业人员30万人，22万下岗失业人员和4万“4050”人员实现再就业，16万下岗职工基本生活保障向失业保险并轨，城镇登记失业率控制在4%以内。财政的社会保障支出逐年增加，确保了企业离退休人员养老金、国企下岗职工基本生活费、失业人员失业保险金、低保群众最低生活费和困难群众临时救济金的按时足额发放。城市居民人均可支配收入和农民人均纯收入分别达到10000元和4180元，比2000年增加4432元和1612元。城市和农村居民恩格尔系数分别下降6和2个百分点。实现城镇居民人均住房建筑面积25平方米，农村人均生活用房使用面积22平方米，城市居民万人拥有私人小汽车624辆，百户拥有移动电话157部、电脑40台。消费结构进一步升级，城市居民文化、教育、旅游人均年支出达到964元。

　　——科技教育与各项社会事业协调发展，精神文明建设全面推进。深入实施科教兴市战略，取得各类科技成果1542项,申请专利9831项。教育事业全面发展，适龄儿童和初中适龄人口入学率均保持在99%以上，实现高中入学率79.5%，高考专科以上进线率96.9%，职业学校毕业生一次性就业率96%。农村办学条件明显改善，累计改造危倒校舍41.6万平方米。中小学普及现代远程教育。建立了突发公共卫生事件防治体系，新传染病医院、四县（市）和双阳区新疾控中心投入使用。稳步推进九台新型农村合作医疗试点，全市141所乡镇卫生院功能基本得到恢复。城区人均体育用地增加到2.03平方米，处于全国同类城市领先水平。文化、广播电视、新闻出版、计划生育等各项事业长足发展。亚冬会筹备工作进展顺利，完成了伪满皇宫二期改造，莲花山滑雪场、长影世纪城等一批项目投入使用。实施人才强市战略，人才总量不断增加，整体素质不断提高。社区服务功能不断完善，援藏工作成效明显，民族宗教工作得到加强。

　　——依法行政不断加强，服务型政府和软环境建设取得新成绩。主动接受人大法律监督、工作监督和政协民主监督，认真办理人大议案、建议、意见和政协建议案、提案，办结率、面复率、满意率逐步提高。加快推进政务公开和电子政务，建立并落实重大决策咨询、政府信息公开和新闻发言人制度，政府工作透明度和行政能力进一步提高。成立集中办理行政许可的政务中心，开展落实《行政许可法》、清理规范性文件、清费减负和“百名处长”评议等多项活动，软环境建设不断加强。扎实推进依法治市，切实加强廉政建设，认真处理群众来信来访，深入开展“严打”斗争，强化安全生产的监督和管理，人民群众的安全感不断增强，社会保持稳定。

　　“十五”期间，长春市先后被授予“国家园林城市”、“国家环境保护模范城市”、“中国人居环境范例奖”、“全国创建文明城市工作先进城市”、“全国社会治安综合治理先进城市”、“全国双拥模范城”、“全国‘两基’先进城市”、“全国职业教育先进城市”、“全国群众体育先进市”、“全国科技进步先进市”、“全国再就业工作先进城市”、 “全国造林绿化先进市”、“全国农业产业化先进单位”、“全国民族团结进步先进集体”、“全国人民防空先进城市”和“全国实施妇女儿童发展纲要先进集体”等一系列荣誉称号。

　　2005年，是“十五”计划的最后一年，也是振兴长春老工业基地的关键一年。一年来，全市上下团结一心，克服汽车产业整体下滑和洪涝灾害等不利影响，坚持加快发展不动摇，各项工作取得了新成绩。一是坚持奋发有为，紧紧抓住振兴老工业基地的关键环节寻求突破，开创了振兴工作的新局面。国企改革取得较大突破，列入省市国企改革攻坚计划的211户企业中，有206户完成方案审批，进入改革操作程序。招商引资成果喜人，引进内资、实际和直接利用外资分别增长23.3%、30%和36.7%。固定资产投资大幅度增加，增长41.3%。二是坚持迎难而上，积极调整优化产业结构，努力培育新的经济增长点。一汽卡车新基地、红旗补充型及换代产品扩建、一汽——大众PQ35平台三大项目建设进展顺利，农产品加工业快速增长，生物医药、光电子信息、物流产业迅速发展，能源、建筑和材料制造、旅游会展、文化、金融产业不断壮大。三是坚持规划先行，全面加强城市建设与管理，城市的知名度和影响力不断提高。完成了新一轮城市总体规划修编，积极调整土地利用总体规划，保证了城市建设和经济发展用地需求。城市发展空间得到扩展，南部新城建设迅速启动，旧城区基础设施改造稳步推进，创建全国文明城市活动取得阶段性成果。四是坚持统筹兼顾，正确处理经济与社会、城市与农村、人与自然的关系，努力实现更快更好的发展。城乡结合部薄弱校改造得到加强，文化体制改革不断深化，集中整治了锅炉烟尘、机动车尾气和污染严重企业，农防林更新改造和水土综合治理步伐加快，县域经济速度与效益同步增长。五是坚持以人为本，认真解决涉及群众利益的突出问题，使全市人民实实在在地享受到改革发展的成果。提高城市居民最低生活保障标准，启动农村最低生活保障制度试点，改造了全市近半数的敬老院，改造巷道1557条，建设乡村公路3296公里，安置2810名残疾人就业，九台营城沉陷区2566户受灾居民迁入新居，建设“平安长春”活动取得明显成效。六是坚持依法行政，进一步规范审批和简政放权，行政体制改革不断向纵深推进。依法整合政务中心审批窗口，全面清理行政许可审批项目，基本建设项目“一次性”收费实现试运行。向四县（市）和双阳区放权197项，开发区享受市级管理权限基本落实到位。

　　各位代表，“十五”时期，是我市经济持续快速健康发展的五年，是社会事业全面进步的五年，是生态环境质量不断提高的五年，也是广大人民群众不断得到实惠的五年。这些成绩的取得，是我们全面贯彻落实中央、省各项方针政策和重大部署的结果，是市委正确领导和市人大、政协有效监督支持的结果，是全市广大干部群众同心协力、顽强拼搏的结果。五年来，海内外朋友、港澳台同胞及外埠工商人士热诚关心、支持、帮助长春的建设和发展。各民主党派、工商联、人民团体和社会各界人士，中央、省驻长单位，中国人民解放军和武警驻长部队全体官兵，为长春三个文明建设作出了巨大贡献。在此，我代表市人民政府表示崇高的敬意和衷心的感谢！

　　回顾过去的五年，我们清醒地看到，长春经济社会发展中还存在不少困难和问题。总量不大、结构不优、效益不好、活力不足，仍然是困扰我市经济社会发展的主要矛盾。特别是受汽车市场变化影响，工业生产增幅和效益有较大幅度下滑，反映出我市产业结构单一，经济整体抗波动能力仍然较弱；农业基础薄弱的状况没有彻底改变，农民增收渠道需要进一步拓宽；就业和社会保障压力较大，困难群众生产生活问题仍需下大气力解决；影响社会安定的因素增多，维护社会稳定任务依然繁重；政府职能转变仍然滞后，办事效率和服务水平不高；个别工作人员为政不廉，等等。对于这些问题，我们将在今后的工作中采取更加有力的措施，认真加以解决。

　　二、“十一五”发展目标和主要任务

　　“十一五”时期，是全面建设小康社会承前启后的关键时期，也是振兴长春老工业基地决战决胜的重要时期，既面临复杂多变的形势和不利因素，更具有诸多有利的发展条件和机遇。我们必须全面增强历史责任感和时代紧迫感，聚精会神抓经济，千方百计快发展，使长春在新的起点上更快更好地发展，逐步实现富民强市的宏伟目标。

　　“十一五”时期，我市国民经济和社会发展的总体思路是：以邓小平理论和“三个代表”重要思想为指导，以科学发展观统领经济社会发展全局，加快转变经济增长方式，继续深入实施依法治市、科教兴市、开放带动、县域突破和可持续发展五大战略，加快建设现代制造业、区域性物流和科教文化三大中心，迅速壮大“两大支柱产业”，大力发展“三大主导产业”，积极培育“五大重点产业”，做大做强开发区、城区和县域三大板块，努力扩大就业，增强经济实力，提高经济抗波动能力和城市综合竞争力。

　　“十一五”时期，我市经济和社会发展的主要预期目标是：全市地区生产总值年均增长13%左右，财政收入按可比口径实现同步增长；地区生产总值能耗降低30%左右；全社会固定资产投资年均增长20%以上；社会消费品零售总额年均增长12%以上；外贸进出口总额年均增长16%以上；实际利用外资年均增长20%以上；城镇登记失业率控制在5%以内；城市居民人均可支配收入、农民人均纯收入年均分别增长9%左右和7%左右。到2010年，基本完成长春老工业基地振兴的历史使命，把长春初步建设成为产业特色鲜明、经济结构合理、服务功能完善、生态环境良好、现代化程度较高的区域性中心城市。

　　实现上述目标，必须坚持更快更好地发展，必须坚持调整优化经济结构，必须坚持提高自主创新能力，必须坚持推进工业化进程，必须坚持深化各项改革，必须坚持全方位对外开放，必须坚持以人为本。突出抓好以下主要工作：

　　（一）不断提升产业竞争力，加快建立多元化产业发展体系。进一步做大做强汽车产业。大力提高自主创新能力、整车生产能力和零部件配套能力，力争2010年一汽集团和一汽——大众合计实现产值2000亿元，汽车零部件及与一汽差异化整车生产实现产值1000亿元，建设国家级汽车生产、研发和出口基地，打造长春国际汽车城。迅速壮大农产品加工业。全力支持大成、皓月、德大等龙头企业快速发展，加快化工醇、赖氨酸、有机醇、淀粉糖、聚乳酸等系列产品项目开发建设，尽快形成玉米和畜牧产品精深加工体系，建设国内重要的农产品加工基地，打造绿色食品城。大力发展光电子信息、生物医药和物流三大主导产业。加快建设国家光电子产业基地，重点发展光显示和汽车电子两大产业链条，拓展光电子器件和材料、光电仪器仪表和设备、软件三大领域。加快建设国家生物产业基地和医药出口基地，重点发展生物制药、现代中药、医药中间体和化学药，扩大生物技术应用领域，加大新药开发力度。加快建设以都市圈物流配送为基础、以物流园区为核心、以综合性和专业性物流配送区为结点、以国际物流为导向的区域性物流中心。积极培育能源、建筑和材料制造、旅游会展、文化、金融五大重点产业，形成多元化产业发展格局。

　　（二）进一步调整城市空间布局，构筑现代化都市发展框架。按照“主副中心、分散组团、轴向发展、带状增长”的发展格局，建设城市西南、

　　东北方向的工业发展轴和城市东南、西北方向的生态建设轴，促进城区发展按照“X”式合理有序展开。坚持城乡统筹规划，加快次中心城市和重点城镇建设，引导产业、人口和其它要素向其集聚。加快建设长吉经济带，与东北其它3个副省级城市共建东北物流走廊和东北经济隆起带。坚持适度超前原则，启动城市快速道路系统一期工程，完善市区交通网络。建设哈大铁路客运专线、铁路客运西站、长白铁路复线和长双烟地方铁路，提升龙嘉国际机场功能，努力构建立体化交通体系。加快水、电、煤炭、燃气资源开发建设，有计划、有组织地完成重点棚户区改造，全面实施伊通河城区段综合治理和改造工程，加快推进城市信息化。发挥比较优势，培育城市特色，全力打造长春国际汽车城、绿色食品城、科教文化城、电影城、森林城、雕塑城，努力提高城市综合竞争力。

　　（三）做强开发区、城区、县域三大板块，努力形成特色鲜明、错位发展、优势互补的经济发展新格局。按照“技术创新、产业集聚、环境超越、集约发展、辐射带动”的思路，把开发区建设成现代制造业的聚集区和现代化新城区，使其继续成为全市推进工业发展的平台和载体，成为抢占科技制高点、拓展产业新领域、带动经济快速发展的“火车头”。鼓励和支持开发区根据各自功能定位，进一步做大做强优势特色产业。

　　积极推动城区率先发展。加快推进工业调整转型。根据各城区优势，创新工业形态，积极发展创新型、研发型、生产服务型产业。围绕综合文化科技中心、交通枢纽中心、空港物流开发三大工程建设，大力发展现代服务业。调整城郊农业结构，大力发展设施农业、生态农业和观光农业，努力建设现代化、生态型、高效益的都市农业示范区。

　　加快社会主义新农村建设。按照生产发展、生活宽裕、乡风文明、村容整洁、管理民主的要求，扎实稳步推进新农村建设。突出工业拉动、城乡互动和科技促动，以农村工业化带动农业产业化，实现县域综合实力显著增强、县级财力大幅增长和农民收入持续增加。培育龙头企业，开发建设一批大项目。建设工业园区，重点发展以轻工产品为主的加工制造业。加快发展现代农业，重点建设500万亩专用玉米、300万亩优质大豆、150万亩绿色水稻、200万亩蔬菜、10万亩中药基地，形成肉牛、奶牛、肉鸡、肉鹅、生猪和鹿业6个龙型经济。发展劳务经济，畅通劳务输出渠道，提高劳动力素质。强化政府对农村的公共服务和科技服务，加强对农民的技能培训。加大农村基础设施投入，加快乡村道路建设，发展农村通信，完善农村电网，逐步解决农村的饮水困难和安全问题。加快发展农村文化教育事业，巩固农村九年义务教育，搞好农村中小学危倒校舍改造。加强农村公共卫生和基本医疗服务体系建设，基本建立新型农村合作医疗制度。

　　(四)加快构建科技创新体系，建设创新型城市。把提高自主创新能力摆在突出位置，按照自主创新、重点跨越、支撑发展、引领未来的要求，把握住我市科技发展的重点领域，确定重点突破专项，突出抓好汽车、玉米、光电子信息、生物医药、新材料、铁路客车等领域的科技创新。抓住重大关键技术，培育创新企业，建设工程研究中心、重点试验室和企业技术中心，加强原始创新、集成创新、引进消化吸收再创新，提高持续创新能力。充分发挥政府的主导作用、市场在科技资源配置中的基础性作用、企业在技术创新中的主体作用、科研机构的骨干和引领作用、大学的基础和生力军作用、科技服务机构的中介作用，进一步形成科技创新的整体合力。坚持贯彻尊重劳动、尊重知识、尊重人才、尊重创造的方针，全面实施人才强市战略，完善人才结构，壮大科技人才队伍。不断增强自强自尊的民族精神和全社会的创造活力，通过理论创新、文化创新，为科技创新提供科学的理论指导、有力的制度保障和良好的文化氛围。实施激励自主创新的各项政策，努力创造有利于提高自主创新能力的法制环境、市场环境和各方面条件。加大财政科技投入的力度，增强政府投入调动全社会科技资源配置的能力，形成多元化、多渠道、高效率的科技投入体系，提高科技资源共享利用的效益。

　　（五）加快转变经济增长方式，构筑生态环境体系。建设节约型城市。节约利用土地、水、能源、原材料，大力推行节约型增长方式，构建节约型产业结构，切实提高资源利用效率。发展循环经济。全面推行清洁生产，大力发展节能、降耗、减污产业，鼓励企业循环式生产，推动产业循环式组合。积极开发资源节约技术，重点研究开发和推广应用资源综合利用、资源节约、资源替代等先进技术，积极发展新能源和可再生能源。加强生态建设和环境保护，改善城乡环境质量，创建人与自然和谐共生、适宜居住和创业的生态城市。在城市规划区内保护与扩建林水生态，加快城郊防护林体系建设。在建成区内实施伊通河绿化、八一水库至西湖绿化改造、主要街路绿化、城市园林绿化、新建居民小区绿化工程。充分利用自然资源优势，建设石头口门水库、新立城水库、双阳、山河、石门山、土顶、土门岭、卢家、光明以及农安西部湿地10个生态保护区。加强环境保护，综合整治大气污染、污水、环境噪声、矿山环境、固体废物，建立以生态保护和治理为重点的生态支撑体系。

　　（六）大力推进体制机制创新，努力提升外向型经济发展水平。加快推进改革，突破影响经济社会发展全局的重大体制性障碍。进一步转变政府职能，积极稳步推进科技体制、行政管理体制、劳动就业体制、事业单位、财税体制、分配制度及农村综合配套等各方面改革，进一步完善市场经济体制，形成有利于转变经济增长方式、有利于全面协调可持续发展的新机制。基本完成竞争性领域国有企业的改制任务，鼓励、支持和引导非公有制经济加快发展，进一步推进全民创业，大力发展民营经济。进一步扩大对内对外开放。把招商与亲商、安商、扶商、富商结合起来，努力营造良好的投资环境，不断优化、壮大利用内外资的结构和规模。积极发展对外贸易，优化出口商品结构，着力提高对外贸易的质量和效益。支持有条件企业“走出去”到境外投资，鼓励境外工程承包和劳务输出。

　　（七）坚持以投资拉动为主要动力，谋划建设一批重大工程项目。把项目建设作为经济提速的突破口，构建主导产业、基础设施、社会事业、生态环境和县域经济五大项目支撑体系。积极推进汽车、轨道车辆、玉米生化、畜产品深加工、交通枢纽中心、综合文化科技中心、空港经济区、城市蓝环、西部造林、污水和垃圾处理等20个重大工程项目建设，带动相关行业快速发展，为长春实现更快更好的发展提供有力支撑。

　　（八）认真解决人民群众最关心、最直接、最现实的利益问题，努力构建和谐社会。坚持以人为本，努力建设“文明长春”、“平安长春”、“信用长春”、“和谐长春”。大力开展精神文明创建活动，不断提高群众的思想道德素质、科学文化素质，进入全国文明城市行列。坚持教育优先，推动基础教育和职业教育加快发展，促进教育公平。完善公共卫生和医疗服务体系，努力提高人民群众健康水平。加强文化事业建设，大力发展文化产业，丰富人民群众精神文化生活。加强妇女儿童工作。不断强化治安防范长效机制，严厉打击破坏经济秩序和各类刑事犯罪，遏制黄赌毒等社会丑恶现象蔓延，努力营造稳定、安全、和谐的社会环境。加大安全生产科技投入，完善应急机制，全面提高各类突发事件的安全防范和处置能力，营造安全的生产生活环境。进一步健全和完善食品安全体系。积极培育信用主体，健全信用机制，完善信用体系，发展信用服务，营造信用环境。进一步拓宽就业渠道，鼓励发展劳动密集型产业、就业容量大的服务业、中小企业和非公有制企业，开发更多的就业岗位。加快建立政府扶持、社会参与的职业技能培训机制，强化政府促进就业的服务职能，健全就业服务体系，促进多种形式就业，鼓励和支持自主创业、自谋职业。完善社会保障体系，合理调解收入分配，推进民主法治进程。加强农民负担监管，保护农民合法权益。

　　三、2006年主要任务

　　2006年是实施“十一五”规划的第一年，也是困难与希望同在、挑战与机遇并存的一年。影响今年经济增长的不确定因素仍然很多，特别受国内外汽车市场激烈竞争和石油价格的影响，汽车产业快速增长的压力仍然较大。因此，必须认清形势，统一思想，加大措施，应对挑战，有效扼制经济增幅和效益下滑趋势，全力保持经济稳步增长、社会全面进步，确保“十一五”实现良好开局。全市地区生产总值增长13%左右，全口径财政收入按可比口径同步增长，城市居民人均可支配收入、农民人均纯收入分别增长10%左右和7%左右。

　　（一）加快调整产业结构，推动工业经济持续稳步发展。坚定不移地举全市之力支持一汽加快发展。全面落实支持一汽的各项政策措施，促进一汽加快分离办社会职能。突出抓好汽车产业开发区建设，全面提高我市整车特别是低速载货车和农机产品生产、专用车生产、零部件配套和技术创新能力，启动天合富奥安全系统等16个汽车及零部件重点项目。汽车工业产值增长15%以上。加快推进农产品加工业。高标准建设玉米工业园，确保20万吨化工醇项目建成投产，玉米工业园基础设施、50万吨化工醇和50万吨聚酯项目开工建设。加快建设天景玉米等69个投资3000万元以上的重点项目，做大做强肉牛、生猪等十大农畜产品产业链。农产品加工业产值增长30%以上。围绕产业集聚、规模发展和扩大国际合作，加快建设长春国家光电子产业和生物产业基地。液晶显示模块、流感疫苗等10个项目建成投产，光电子信息和生物医药产业产值均增长30%左右。全面加强能源、建筑和材料制造等具有先导和支撑作用的基础产业，热电三厂等一批骨干项目建设取得实质性进展。坚持以信息化带动工业化，广泛应用高新技术和先进适用技术改造提升传统产业。实施名牌带动战略，不断推动名优产品扩大规模、增加效益，新创省级以上名牌30个。规模以上工业实现产值1970亿元左右。

　　（二）加快农村经济社会发展，扎实推进社会主义新农村建设。进一步提高粮食综合生产能力，优化种植业结构，加快建设各类优质原料基地，专用玉米、优质大豆和绿色水稻分别发展到200万亩、150万亩和100万亩。加快发展畜牧业，新建标准化牧业小区100个，畜牧业产值增长6%以上。加速推进农村劳动力转移，农民外出务工经商95万人次。大力促进农业产业化经营，订单农业面积达到800万亩，各类农村新型经济组织发展到1000个。重点建设五棵树、米沙子等14个工业集中区，启动实施投资超千万元的工业项目100个。深化农村乡镇机构和财政体制改革，巩固农村税费改革成果。增加农村教育、文化、卫生等方面的投入，推动农村社会事业全面进步。继续改造乡镇卫生院，启动榆树、农安新型合作医疗试点。实施农村最低生活保障制度。加大农村基础设施投入，提升农田基本建设、农村道路、饮水、电网、通信等建设水平。搞好中小型水库除险加固，实施长春经济环线公路工程，新建乡村公路1000公里，基本实现村村通油（水泥）路。

　　（三）扩大投资、促进消费，拉动经济快速增长。围绕“十一五”规划，超前谋划和启动一批带动作用强、辐射范围大、发展后劲足的大项目。加强组织协调，广开资金渠道，培育投资主体，尽快形成投资主体多元化、资金来源社会化、投资方式多样化、项目建设市场化的新格局，力争全社会固定资产投资增长30%以上。抓紧修编土地利用总体规划，加大土地收储力度，提高土地经营水平，确保重点项目用地需求。

　　更加注重扩大消费需求，充分发挥消费对经济增长的拉动作用。鼓励发展新型业态，完善商业网点布局，促进市场繁荣稳定。社会消费品零售总额增长12%以上。加快建设汽车等4大物流园区和生产资料、生活资料、农产品等8大物流配送中心，大力培育和引进物流骨干企业。物流业营业收入增长20%以上。积极发展旅游会展、文化、金融等需求潜力大、成长能力强的服务业，培育新的经济增长点。办好冰雪节、农博会、东博会、电影节、雕塑展等21项重点展会，全年举办各类会展活动120项以上。下力气引进和发展各类金融机构，活跃金融市场，逐步完善适应加快发展需要的金融支撑体系。进一步整顿规范市场经济秩序，坚决打击假冒伪劣和经营欺诈行为，维护企业和消费者权益。

　　（四）坚定不移地深化改革、扩大开放，进一步增强经济社会发展的活力和动力。全面深化各项改革。继续推进国有大型企业股份制改造，加快建立现代企业制度。妥善处理改制企业遗留问题，制定和落实扶持政策，支持改革后企业加快发展步伐，尽快做大做强。加快推进粮食流通体制改革，继续提高粮食购销的市场化程度。深化市属事业单位人事制度改革，启动县（市）区事业单位人事制度改革。进一步推进文化体制改革，经营性文化事业单位实现“事转企”，专业艺术院团改革迈出实质性步伐。积极稳妥地推进国有资产管理体制、投融资体制、科技体制等方面改革，力争在一些重点领域和关键环节上取得突破。

　　努力扩大对外开放。突出引资重点，创新引资方式，打造引资平台，努力提高利用内外资的规模和质量。利用内、外资均增长20%以上。大力开拓国际市场，优化出口商品结构，重点增加汽车及零部件、农畜产品深加工、医药产品出口。启动建设空港经济区，新开通国内外航线10条以上。加快推进对内放开。认真落实扶持民营经济发展的各项政策，大力发展和提倡个人经济、小本经济、楼宇经济，积极打造创业基地、创业街路、创业市场等创业平台，培育创业文化，推动全民创业。建立健全服务体系，帮助民营企业解决技术、资金等实际困难。支持有实力的民营企业迅速扩张，扶持一批民营科技企业进入“小巨人”行列。民营经济增加值增长20%左右。

　　积极推进开发区二次创业。全面加快开发区的创新和发展步伐，力争各开发区在体制机制创新、软环境建设、拓展空间、招商引资、减轻负担、提速增效、建设和谐区域、形成合力8个方面实现新的突破。开发区实现生产总值1000亿元，引进超千万元项目130个以上，固定资产投资增长30%以上。

　　（五）全面加强城市建设与管理，努力提高城市吸引力和承载力。在城市总体规划指导下，高标准编制分区规划、专项规划、控制性详细规划和村镇规划，强化规划监管，提高规划的编制和审批效率。实施“十路四桥两出口”建设工程，加快汽车产业开发区、玉米工业园、铁北及南部新城核心区路网建设。继续搞好城区主次干道大中修，改造巷道1000条。继续推进房地产业发展，改造棚户区240万平方米。改造供水管网50公里、燃气管网50公里、旱厕100个，集中亮化296条背街暗巷。启动城市交通枢纽工程，轻铁二期投入使用。继续改造南湖公园、胜利公园、儿童公园，完善雕塑公园二期，改建裕华园。城区新增绿地300公顷。继续实施“串湖”综合治理，启动东莱明沟改造。加快伊通河改造工程建设，完成卫星桥至四化桥全线14公里蓄水等“四项任务”。建设南部污水处理厂、北郊污水处理厂二期、三间村粪便处理场、蘑菇沟屯垃圾处理场，提高污染物处理能力。推进“三北”防护林四期工程建设和宜林荒山荒地造林改造，完成植树造林5000公顷。力争启动石头口门水库饮用水源地保护工程。进一步强化管理措施，改革与创新城市管理体制，逐步建立起加强城市管理的长效机制。加强城市管理综合行政执法，搞好市容环境综合整治。深入开展创建国家卫生城市活动，全市2/3以上社区达到“卫生先进社区”标准。积极推进清洁生产，加强资源综合利用，地区生产总值能耗降低6%左右。

　　（六）坚持科教兴市和人才强市战略，大力发展社会事业。努力增强自主创新能力，积极实施政府科技发展计划，加大政府引导力度，加快以企业为主体的高技术和科技成果的开发、转化、产业化，高新技术产值增长20%以上。推进中俄、中德科技合作基地建设，启动建设中欧合作长春工业园区。加强知识产权保护。大力开发人才资源，切实强化高层次和紧缺人才队伍建设，引进高层次及各类急需紧缺人才1.2万人，培训公务员和企业经营管理、专业技术、高技能、农村实用人才23万人次。

　　巩固义务教育成果，提高高中办学质量，大力发展职业教育。维修改造5所城乡结合部薄弱学校，四县（市）和双阳区各建1个1500至4000人规模的职教中心。完善公共卫生和医疗服务体系，控制艾滋病、人间禽流感等重大传染病。稳定人口低生育水平，提高出生人口素质。加大政府对公益性文化事业的投入，逐步形成覆盖全社会、比较完备的公共文化服务体系。做好群众体育、广播电视、新闻出版等各项工作。启动建设综合文化科技中心。

　　以创建全国文明城市为目标，大力加强社会主义精神文明建设。深入开展双拥活动，争创全国双拥模范城“六连冠”。加强社会治安综合治理，依法严厉打击各种犯罪，保障人民群众生命财产安全。完善社会治安防控体系，提高维护公共安全和处置突发事件能力。进一步推进食品放心工程，强化危险化学品和交通安全监管，防止重特大事故发生。注重城市安全，提高处置突发事件能力。

　　全市动员、全民参与，高标准完成亚冬会各项筹备工作。认真落实《亚冬会行动计划》，力争把2007年长春亚冬会办成最成功、最具魅力的冬季体育盛会。充分挖掘亚冬会的综合效应，带动经济社会发展，全面提升城市综合服务功能和整体形象。

　　（七）努力扩大就业，完善社会保障体系。努力把扩大就业摆在经济社会发展更加突出的位置，深入开展创业促就业活动，加大再就业援助力度，增加资金投入，落实优惠政策，千方百计扩大就业。全年新增就业岗位9万个，安置下岗失业人员6万人，其中“4050”人员1万人。增加财政社会保障投入，依法加大社会保险基金征缴力度，进一步扩大养老、失业、医疗、工伤、生育保险覆盖面，抓好非公经济组织和事业单位参保工作，逐步将符合条件的城镇从业人员和失地农民纳入社会保险范围。

　　下力气改善困难群众生活。扩大廉租房差额面积补贴范围，妥善安置低收入拆迁户住房，进一步扩大低价位商品房开发比例，逐步解决困难家庭住房问题。规范医疗药品收费，进一步解决群众看病难、医药贵问题。公办义务教育阶段学校严格实行“一费制”，继续对城镇贫困户子女入学给予资助。基本完成农村敬老院改造任务。大力发展慈善事业，加快社会救助体系建设。

　　（八）加强民主法制建设，努力建设服务型政府。全面实施《依法行政纲要》，坚持依法行政、依法决策、依法办事。认真贯彻人大及其常委会决议，完善工作报告制度，自觉接受人大的法律监督、工作监督和政协的民主监督。切实做好人大代表建议、批评、意见和政协提案的办理工作，为各民主党派、无党派人士和市民参政议政创造条件。完善公众参与、专家论证与政府决策相结合的民主决策机制，严格执行决策程序。落实行政执法责任制和过错追究制，加强对行政执法行为的监督和规范。扩大基层民主，落实村务公开，搞好社区建设。完善信访工作责任制，及时处理信访问题，妥善处置群体性事件。

　　大力加强服务型政府建设。进一步推进政企分开、政资分开、政事分开。在抓好经济调节、市场监管的同时，更加注重社会管理和公共服务，使政府服务走出机关，重心下沉，积极主动地为基层、企业、群众解决实际困难和问题。采取各种有效措施，狠抓各项工作落实。建立和完善重点工作目标责任制，全面加强督促检查，严格兑现奖惩，切实提高行政效能。重实干，办实事，求实效，着力改进政风。贯彻落实《公务员法》，努力建设高素质的公务员队伍。

　　 进一步加强软环境建设。把软环境建设提升到社会信用体系建设新阶段，建设诚信政府，逐步建立社会信用监控体系和失信惩戒机制。进一步推进政务公开，继续完善政务中心服务功能，建立并试行振兴老工业基地项目、重大招商引资项目行政审批全程无偿代办制。逐步完善行政许可网上办理功能。继续开展民主评议政行风和“百名处长”评议活动，深入清理行政事业性收费，严肃查处涉软案件。坚持廉洁从政，加强廉政建设和反腐败斗争，严格落实领导干部经济责任审计制度、建设工程招投标制度、政府采购制度，从源头上预防和治理腐败，建设节约型政府。

　　各位代表！回首五年成就，令人鼓舞；展望未来发展，催人奋进。让我们更加紧密地团结在以胡锦涛同志为总书记的党中央周围，高举邓小平理论和“三个代表”重要思想伟大旗帜，在市委的正确领导下，在市人大、政协的监督和支持下，齐心协力，埋头苦干，为振兴长春老工业基地，为实现全面建设小康社会和“十一五”规划的宏伟目标而努力奋斗！
